# Human Bone Marrow-derived Mesenchymal Stem Cell: A Source for Cell-Based Therapy

**Published:** 2012-02-01

**Authors:** M. Ayatollahi, B. Geramizadeh, M. Zakerinia, M. Ramzi, R. Yaghobi, P. Hadadi, A. R. Rezvani, M. Aghdai, N. Azarpira, H. Karimi

**Affiliations:** 1 *Transplant Research Center, Shiraz University of Medical Sciences, Shiraz, Iran.*; 2 *Hematology Research Center and Bone Marrow Transplantation Unit, Shiraz University of Medical Sciences, Shiraz, Iran.*

**Keywords:** Mesenchymal stem cells, Human bone marrow, cell therapy

## Abstract

Background: The ability of mesenchymal stem cells (MSCs) to differentiate into many cell types, and modulate immune responses, makes them an attractive therapeutic tool for cell transplantation and tissue engineering.

Objective: This project was designed for isolation, culture, and characterization of human marrow-derived MSCs based on the immunophenotypic markers and the differentiation potential.

Methods: Bone marrow of healthy donors was aspirated from the iliac crest. Mononuclear cells were layered over the Ficoll-Paque density-gradient and plated in tissue cultures dish. The adherent cells expanded rapidly and maintained with periodic passages until a relatively homogeneous population was established. The identification of adherent cells and the immune-surface markers was performed by flow cytometric analysis at the third passage. The *in vitro* differentiation of MSCs into osteoblast and adipocytes was also achieved.

Results: The MSCs were CD11b (CR3), CD45, CD34, CD31 (PCAM-1), CD40, CD80 (B7-1), and HLA-class II negative because antigen expression was less than 5%, while they showed a high expression of CD90, and CD73. The differentiation of osteoblasts, is determined by deposition of a mineralized extracellular matrix in the culture plates that can be detected with Alizarin Red. Adipocytes were easily identified by their morphology and staining with Oil Red.

Conclusion: MSCs can be isolated and expanded from most healthy donors, providing for a source of cell-based therapy.

## INTRODUCTIN

The mesenchymal stem cell (MSC) is one of the most interesting types of the adult stem cell. The primary source of MSCs in adults is the bone marrow, where they are immersed in the stroma [1]. They are present at a low frequency in bone marrow, and recent studies suggest that in humans there is one MSC per 34,000 nucleated cells [2]. Apart from the bone marrow, MSCs are also located in other tissues of the human body such as peripheral blood, cord blood, fat, brain, thymus, muscle, liver, and lung [3].

Since the first description of MSCs by Fridenstein, *et al*, in 1976 as the clonal, plastic- adherent cells [4], the interest in MSCs rapidly grew with expanding knowledge about their exceptional characteristics and usefulness in the cell therapy applications [5,6]. Mouse is an often-used model species for a wide range of cell therapy experiments. The MSCs obtained from rats, rabbits, pigs, and sheep have also been proved for the development of engineered tissues using autologous cells [7]. 

The MSCs may participate also in human cell therapy protocols. Several clinical case reports (reviewed in Kassem, *et al* [8]) have concluded the successful treatment of bone and cartilage defects, vascular ischemia and coronary artery disease, and of chronic skin wounds by local administration of MSCs to the site of injury. The injected cells were well tolerated and some spectacular healing results were obtained [9]. These results indicated that the immunosuppressive effects should be considered whenever MSC transplantation takes place.

Furthermore, there are studies that report the differentiation of MSCs into other cell types [10,11]. The ability of MSCs to differentiate into many cell types, as well as their immunomudulatory effects, make them an attractive therapeutic tool for cell transplantation and tissue engineering.

Despite diverse and growing information on MSCs and their use in cell-based strategies, the mechanisms that govern MSC self-renewal and multilineage differentiation are not well understood and has remained an active area of investigation. 

Moreover, the identification of MSCs is not well known. Cultured MSCs have been extensively analyzed by both morphologic and immunologic characterization. 

A great number of surface markers have been described for committed mesenchymal progenitors [12], and Barry, *et al*, [13] have compiled a long list of candidate markers including CD44, CD29, and CD90, to define of human MSCs. The expression of CD34 is not clearly defined in murine MSCs, but the marker is known to be absent from human and rat cells. More specific antigens, such as CD271, are also important markers for MSCs [14].

Although, there is a great deal of experiments on MSC characterization, none of these characteristics is specific enough to adequately define this cell type [2]. Therefore, research efforts focused on biological and phenotypic characteristics of this highly useful stem cell type are crucial. 

The present study describes a simple method for isolation and rapid expansion of MSCs obtained from human bone marrow, based on immunophenotyping and differentiation potential, providing these cells as a source of stem cell transplantation. 

## MATERIALS AND METHODS

Isolation and culture of human MSCs 

This study was approved by Shiraz University of Medical Sciences Ethics Committee. Bone marrow aspirates (5 mL) were obtained from the iliac crests of human bone marrow donors. The participants were bone marrow donors aged between 19 and 42 years who attended Bone Marrow Transplantation Center, Nemazi Hospital, Shiraz, southern Iran.

Each sample of aspirate was diluted 1:1 with Dulbecco’s modified Eagle’s medium (DMEM)-low glucose (1000 mg/L) (Invitrogen, Merelbeke, Belgium) and layered over about 5 mL of ficoll (Lymphoprep; Oslo, Norway). The isolation method was according to a previously reported method [ 15 ] with some modifications which will be mentioned completely. After centrifugation at 939 g for 20 min, the mononuclear cell layer was removed from the interface. Cells were suspended in DMEM and centrifuged at 338 g for 15 min, then resuspended in basal DMEM medium contained 10% fetal calf serum (Invitrogen, Merelbeke, Belgium), 1% penicillin (Invitrogen, Merelbeke, Belgium), 1% streptomycin (Invitrogen, Merelbeke, Belgium), and 2 mM glutamine (Invitrogen, Merelbeke, Belgium). The cells were seeded at a density of 80,000/cm^2^ in 25 cm^2 ^T-flasks and maintained at 37 °C in an atmosphere of 5% CO_2_. After four days, the non-adherent cells were removed and the media was changed every three days. To expand the MSCs cells, the adhered monolayer was detached with trypsin-EDTA (Invitrogen, Merelbeke, Belgium) for 5 min at 37 °C, after 14 days for the first passage and every 4–5 days for successive passages. During *in vitro *passaging, the cells were seeded at a density of 5–10×10^3^ cells/cm^2^ and expanded for several passages until they no longer reached confluence.

Characterization of MSCs 

At each passage, the cells were counted and analyzed for viability by trypan blue staining analysis. Cultured MSCs have been analyzed both morphologically and with respect to surface markers. Functional ability of differentiation into osteocyte and adipocyte was achieved in response to specific culture conditions. Each experiment described in here was replicated for three times.

Flow cytometric analysis 

The identification of adherent cells was performed by flow cytometric analysis. At the third passage, the cells were detached from the culture flasks with trypsin-EDTA and counted. About 1×10 ^6^ cells were incubated on ice for 30 min with goat serum, resuspended in phosophate-buffered saline (PBS) and pelleted by centrifugation for 4 min at 1035 g. Subsequently, the cells were stained for 30 min at 4 °C with fluorescent isothiocyanate (FITC)-coupled or phycoerytrin (PE)-conjugated CD11b (CR3), CD45, CD34, CD31 (PCAM-1), CD90, CD73, CD40, CD80 (B7-1), and HLA-class II. The labeled cells were thoroughly washed with PBS and analyzed on a flow cytometer (FACS Calibur Becton, Dickinson, USA), using WinMidi software (Scripps Research Institute; San Diego, USA). An isotype control with FITC or PE-labeled was included in each experiment, and specific staining was measured from the cross point of the isotype with the specific antibody graph.

Flow cytometric analysis 

For osteogenic differentiation, the 4th-passage cells were treated with osteogenic medium for three weeks with medium changes twice weekly. Osteogenic medium consisted of DMEM supplemented with 10 ^-8^ M/L dexamethasone (Sigma-Aldrich, St. Louis, USA), 10 mmol/L glycerol phosphate (Sigma-Aldrich, St. Louis, USA), 3.7 g/L sodium bicarbonate (Sigma-Aldrich, St. Louis, USA), and 0.05 g/L ascorbic acid (Sigma-Aldrich, St. Louis, USA). Osteogenesis was assessed by Alizarin Red staining.

Adipogenic differentiation 

To induce adipogenic differentiation, the 4th-passage cells were treated with adipogenic medium for three weeks. Medium changes were performed twice weekly [18]. Adipogenic medium consisted of DMEM supplemented with 1 M/L hydrocortisone (Sigma-Aldrich, St. Louis, USA), 0.05 g/L ascorbic acid, 0.05 g/L indomethacin (Sigma-Aldrich, St. Louis, USA), and 10^-6^ M/L dexamethasone. Adipogenesis was assessed by oil red O staining.

## RESULTS

Isolation and Expansion of human MSCs 

**Figure 1:**
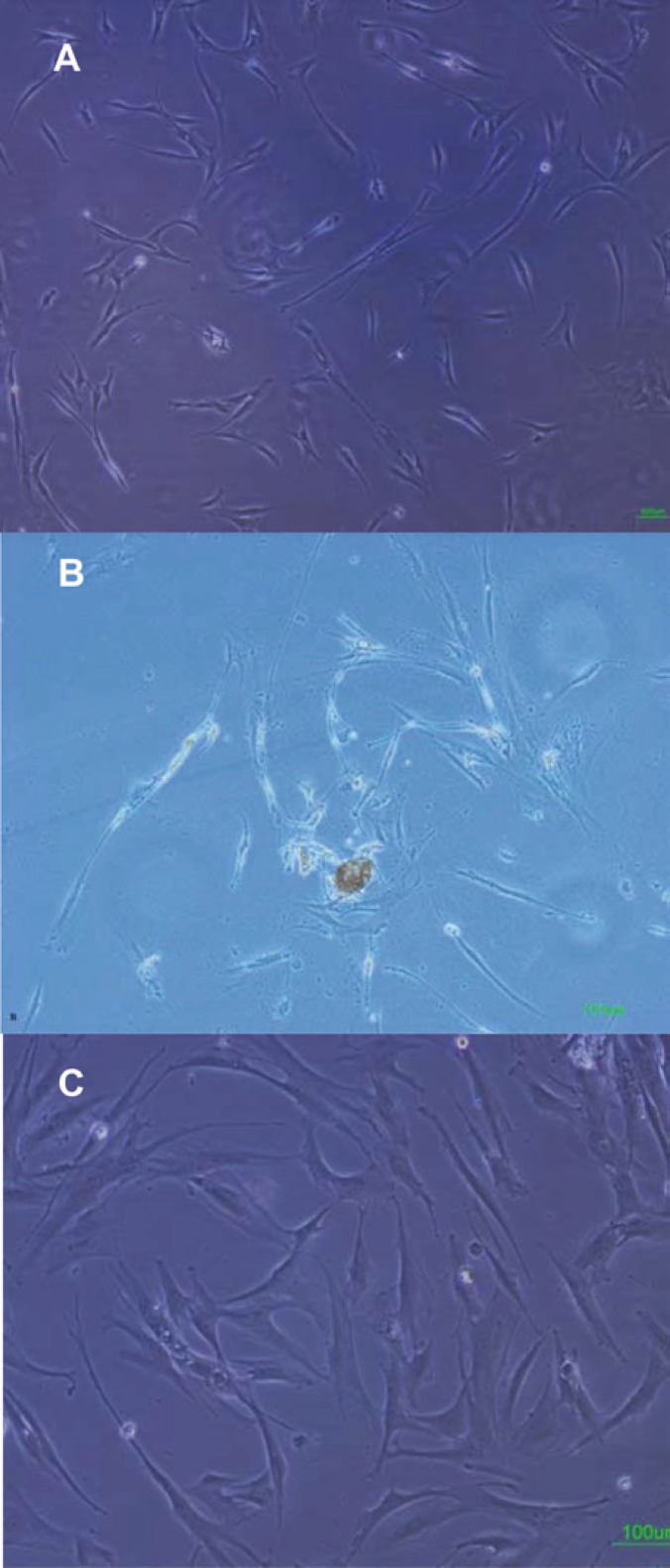
Isolation and culture of human bone marrow derived MSCs. A) adherent monolayer was achieved in the following 6–7 days; B) The presence of single cell-derived colonies composed of a few fibroblast-like cells; C) As the culture proceeded, the cells were both small spindle, and wide-shaped morphology. Scale bar for figures A–C: 100 μm

Adherent cells were observed in all samples after three days culture; within the following 15 days an adherent monolayer was achieved (Fig 1A). The rapid expansion of the MSCs in culture was found to depend on the presence of single cell-derived colonies composed of a few fibroblast-like cells (Fig 1B). Bone marrow cells rapidly generated a confluent layer of cells possessing an elongated, fibroblastic shape. These cells consisted of two cell types: cells with large and flat morphology, and smaller spindle-shaped cells (Fig. 1C). The cells increased in size and showed a polygonal morphology with evident filaments in the cytoplasm especially when early passage cells were compared with late passage cells. MSCs isolated from healthy donors were expanded for up to 10 passages.

Viability evaluation 

At each passage, the cells were counted and analyzed for viability by trypan blue staining analysis showing viability between 98% and 100% in samples. 

Flow cytometric analysis 

**Figure 2:**
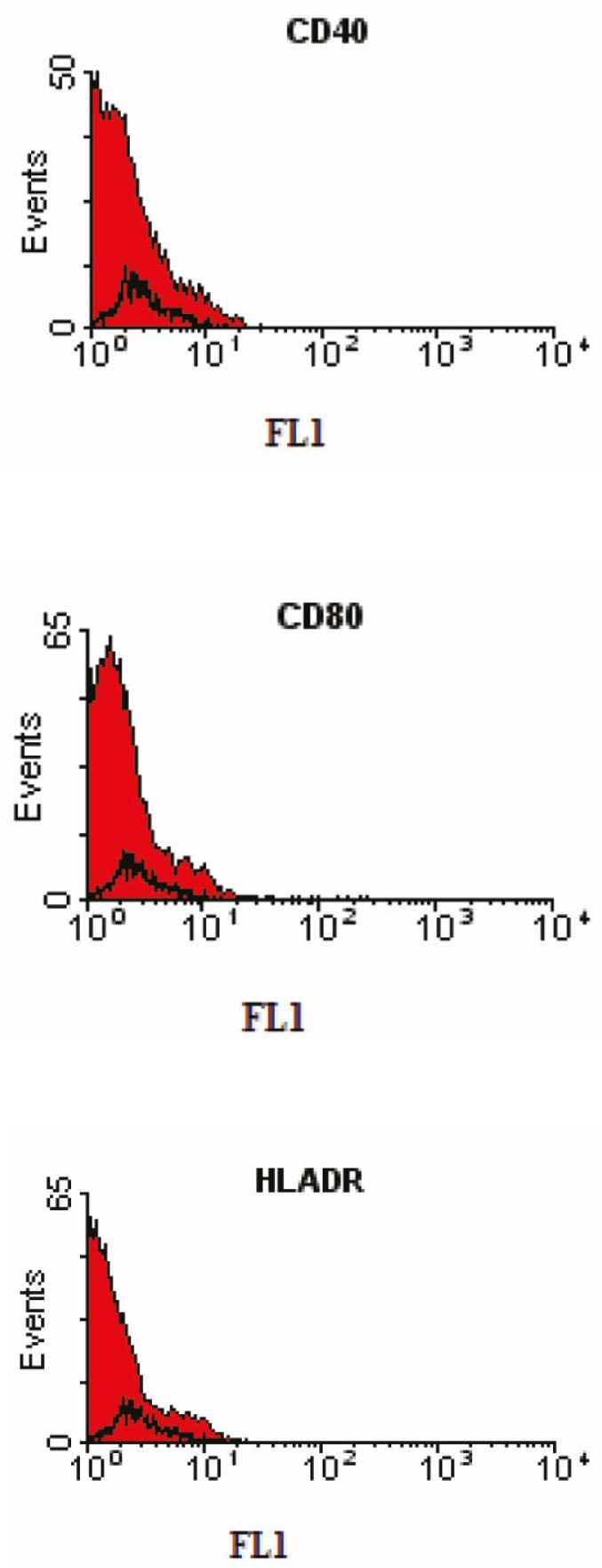
Immunophenotyping of human bone marrow derived MSCs using ﬂow cytometry. The black histogram shows the profile of the isotype control. The cells were negative for CD40, CD80, and HLA-DR.

**Figure 3:**
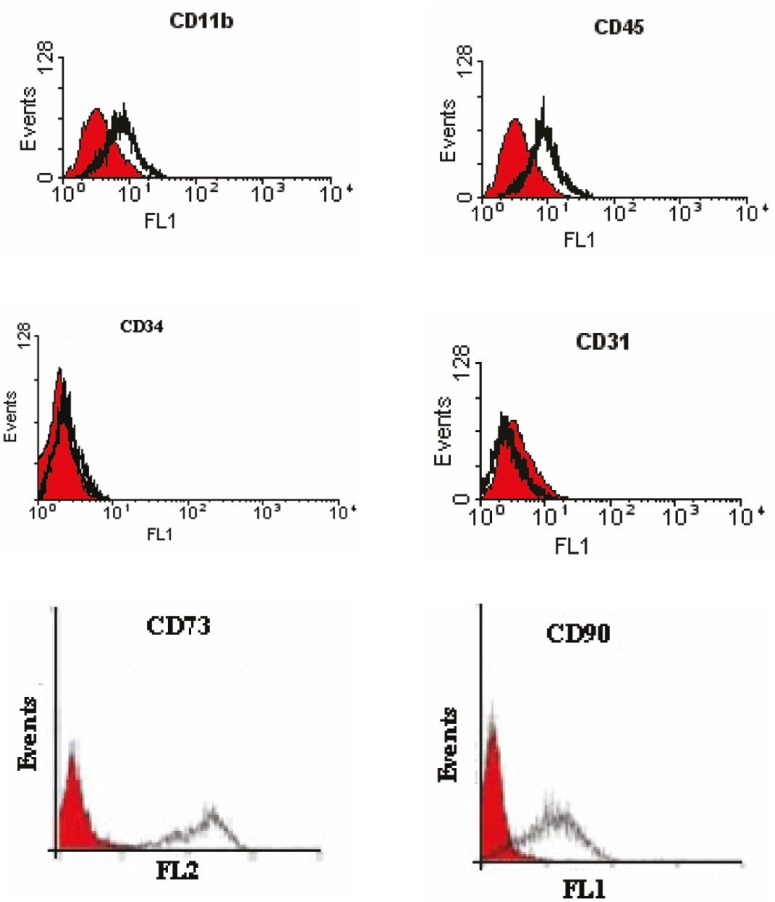
Identification of human bone marrow derived MSCs using ﬂow cytometry. The shaded area shows the profile of the negative control. Mesenchymal stem cells were negative for CD11b, CD45, CD34, and CD31. The cells were positive for CD90 and CD73

We have characterized a population of cells residing human bone marrow cultures that were negative for the major histocompatibillity complex (MHC) class II, CD40, and CD80 (B7-1) costimulators for T lymphocyte activation (Fig 2). Furthermore, no cells expressed the hematopoietic markers CD45, CD34, CD11b (complement receptor), and CD31 (PECAM-1). However, they were positive for the MSC-candidate markers CD90 and CD73 (Fig 3).

Osteogenic differentiation 

**Figure 4:**
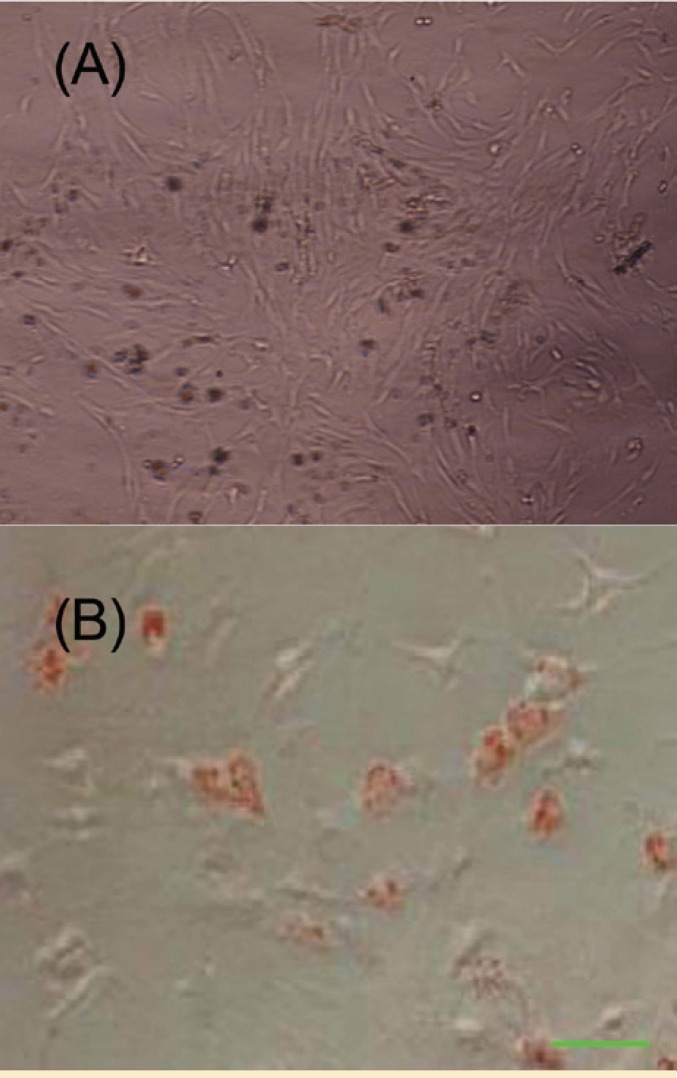
Osteogenic and adipogenic differentiation of bone marrow mesenchymal stem cells. A) Osteogenic differentiation was positive for Alizarin Red staining; B) The adipose droplet in differentiated cells after staining with oil red.

The differentiation of MSCs to osteoblasts *in vitro* involved incubating a confluent monolayer of MSCs with the osteogenic media for 2–3 weeks. The MSCs formed aggregates or nodules, and calcium accumulation could be seen over time. These bone nodules were stained positively by Alizarin Red technique (Fig 4A). 

Adipogenic differentiation 

To promote adipogenic differentiation, MSC cultures were incubated with adipogenic media. An accumulation of lipid-rich vacuoles within cells was seen after three weeks. Eventually, the lipid vacuoles combined and filled the cells. Accumulation of lipid in these vacuoles was assayed histologically by oil red O staining (Fig 4B).

## DISCUSSION

The MSCs may participate in human and animal cell therapy protocols through two mechanisms. First, MSCs may contribute physically to injured sites when administrated locally or systemically. Second, MSCs may have a supportive role by several factors. *In vitro* experiments in which activated murine splenocytes were co-cultured with CH310T1/2 cells in a trans-well culture system indicated that soluble factors secreted by MSCs inhibit CD8+ T cell proliferation [16]. The mechanisms involved in MSC-mediated immunosuppression are currently being investigated. Several studies have demonstrated the immunosuppressive effect of the MSCs in allogenic or mitogenic interactions. Glennie, *et al*, reported that MSCs suppress T cell effector function transiently, but the cells do not block activation [17]. On the other hand, they induce an irreversible proliferation arrest not only in CD4+ and CD8+ T cells, but also in B cells, by down-regulating cyclin-D2 expression [17].

In this study, we have analyzed the immune-surface marker of the human cells in the third passage. They were negative for CD40, CD80 (B7-1), and HLA class-II. CD40 is expressed on B cell, macrophages (MQ), dendritic cells (DC), and endothelial cells (ED). CD40 also bind CD154 (CD40 ligand) and has a role in T cell-dependent B cell activation, and MQ, DC, and ED cell activation. CD80 is costimulator for T lymphocyte activation and is a ligand for CD28 and CD154 (CTLA-4). The main cellular expression of CD80 are DC, activated B cells, and MQs. All these markers can be recognized by alloreactive T cells. Additionally, more than 90% of the mesenchymal cells in our study did not express HLA class-II. These results confirm the low immunogenicity of these cells which should be considered for MSC transplantation. The interactions of MSCs and immune cells may have future implications not only for the knowledge of MSC biology, but also for the understanding of immune system homeostasis. Furthermore, the cells were negative for the hematopoietic markers CD45, CD34, CD11b (complement receptor), and CD31 (platelet/endothelial cell adhesion molecule [PECAM-1]) the common markers of hematopoietic stem cells indicating lack of any hematopoietic cell contamination. However, they were positive for CD90 and CD73 (the MSC-candidate markers) (Fig 3).

The *in vitro* differentiation of MSCs into several lineages in this study was also achieved. Determination of the phenotype of differentiated cells depends on morphological and functional criteria. Alizarin Red staining confirmed the presence of calcium deposits in osteocytes, whereas undifferentiated MSCs were negative in staining procedure. Likewise, lipid droplets in differentiated adipocytes were stained by oil red O staining (Fig 2A-B). These results indicate that the isolated cells have the basic properties of the MSCs.

The *in vitro* cultured MSCs show great heterogeneity in their differentiation potential. Although the analysis of established MSC cultures show them to be pluripotent, with a tri-lineage (osteo-/chondro-/adipo-) [18] or even higher differentiation potential and great plasticity [19], but these cells are poorly defined. This has led to heterogeneity in their sources, phenotypes and multidifferentiation potential. In our previous study, we have developed a novel protocol for hepatic differentiation of human marrow-derived MSCs based on a combination of IGF-I with the growth factors hepatocyte growth factor (HGF), and oncostatin M [10]. The present study describes a simple method for isolation and rapid expansion of MSCs from human bone marrow which confirms the differentiation potential of these cells into mesodermal lineage as well as immunophenotype characterization. The MSCs isolated and expanded in this study, expressed some mesenchymal lineage surface epitopes, grew as adherent cells and successfully differentiated into bone and adipose cells within a short period. Thus, large quantities of human MSCs can be generated for potential cell therapy or tissue engineering applications.
